# Six decades of warming and drought in the world’s top wheat-producing countries offset the benefits of rising CO_2_ to yield

**DOI:** 10.1038/s41598-022-11423-1

**Published:** 2022-05-13

**Authors:** David Helman, David J. Bonfil

**Affiliations:** 1 Institute of Environmental Sciences (Soil and Water Sciences), The Robert H. Smith Faculty of Agriculture, Food and Environment, The Hebrew University, 7610001 Rehovot, Israel; 2 Advanced School for Environmental Sciences, The Hebrew University, Jerusalem, Israel; 3 Department of Vegetable and Field Crop Research, Agricultural Research Organization, Gilat Research Center, 8531100 Beit Dagan, Israel

**Keywords:** Carbon cycle, Climate change, Climate-change ecology, Agroecology

## Abstract

Future atmospheric carbon-dioxide concentration ([CO_2_]) rise is expected to increase the grain yield of C3 crops like wheat even higher under drought. This expectation is based on small-scale experiments and model simulations based on such observations. However, this combined effect has never been confirmed through actual observations at the nationwide or regional scale. We present the first evidence that warming and drought in the world’s leading wheat-producing countries offset the benefits of increasing [CO_2_] to wheat yield in the last six decades. Using country-level wheat yield census observations, [CO_2_] records, and gridded climate data in a statistical model based on a well-established methodology, we show that a [CO_2_] rise of ~ 98 μmol mol^−1^ increased the yield by 7% in the area of the top-twelve wheat-producing countries, while warming of 1.2 °C and water depletion of ~ 29 mm m^−2^ reduced the wheat grain yield by ~ 3% and ~ 1%, respectively, in the last six decades (1961–2019). Our statistical model corroborated the beneficial effect of [CO_2_] but contrasted the expected increase of grain yield under drought. Moreover, the increase in [CO_2_] barely offsets the adverse impacts of warming and drought in countries like Germany and France, with a net yield loss of 3.1% and no gain, respectively, at the end of the sampling period relative to the 1961–1965 baseline. In China and the wheat-growing areas of the former Soviet Union—two of the three largest wheat-producing regions—yields were ~ 5.5% less than expected from current [CO_2_] levels. Our results suggest shifting our efforts towards more experimental studies set in currently warm and dry areas and combining these with statistical and numerical modeling to improve our understanding of future impacts of a warmer and drier world with higher [CO_2_].

## Introduction

The atmospheric concentration of carbon dioxide ([CO_2_]) has been increasing rapidly since pre-industrial times from a steady 280 to 415 μmol mol^−1^ currently and is expected to reach ∼550–600 μmol mol^−1^ by the middle of this century^1^. Continuous elevation in [CO_2_] level and more frequent and intense heatwaves and droughts, already observed in many agricultural areas^2^, are expected to have a tremendous impact on crops, with significant implications for global food security^3,4^.

Wheat, staple food and one of the four major crops cultivated worldwide, may be dramatically affected by such drastic environmental and climatic changes^5,6^. Climate variability was shown to account for $\sim$ 35% of global wheat yield variation, with differences between cold and warm regions^7^. Global warming has resulted in large variations in wheat yield, with expected losses reaching up to 6.4% for each 1 °C rise in temperature^8,9^. Frequent droughts involving many cropping areas are expected to increase the risk of wheat yield loss by almost 12% by the end of the twenty-first century^10^.

While the effects of warming and drought on wheat (and other crops) have been documented at various scales, the interactive effect of [CO_2_] and climate has yet to be elucidated. Most of our understanding of the mechanisms underlying the effects of [CO_2_] on crops is based on experimental manipulations, including crop exposure to [CO_2_]-enriched environments under various temperature and water conditions. The experiments may be performed using plants grown in pots under greenhouse conditions. However, more natural and larger-scale experiments may involve open-top-chambers (OTC) or free-air carbon dioxide enrichment (FACE) experiments^11^. The primary conclusion from almost all such studies is that C3 crops (such as wheat) increase their yield under elevated [CO_2_]^12–15^. The increase in yield is mostly via enhanced net carbon assimilation rate (*A*)^16^, decreased stomatal conductivity^17,18^, and reduced transpiration^19^, which improves crop water productivity, defined as the ratio of crop yield to its water use^20^. Thus, under elevated CO_2_ C3 crops such as wheat are expected to increase their relative yield even higher under drought^21^, though this does not necessarily mean absolute yield gain. Moreover, an increase in yield under different water regimes may affect growth processes such as grain protein content differently^22^.

Though some have challenged this beneficial effect of [CO_2_] on soil water saving and yield under dry conditions^23^, current models use the aforementioned mechanisms to simulate crop response to future [CO_2_] and climate scenarios on the regional and global-scale^24–27^. After years of simulating crop response to warming and drought without considering the effect of [CO_2_], modelers have been recently called to include such an effect claiming that “uncertainties in the effects of elevated [CO_2_] on crops have narrowed”^28^. However, a few FACE sites have studied the benefits of elevated [CO_2_] under warming and drought, and the effects have yet to be validated on a larger scale.

Here, we analyze nationwide census wheat yield data obtained from the world’s largest wheat-producing countries from 1961 to 2019 to assess the individual and combined effects of climate and [CO_2_] on wheat yield. Leveraging six decades of recorded atmospheric [CO_2_] data and census observations of country-level yields, we provide estimates of yield variation due to temperature, water deficit, rainfall distribution, and [CO_2_], showing that the beneficial effect of [CO_2_] on wheat yield diminishes under increased water deficit conditions, with major losses during the last six decades occurring mainly in leading wheat-producing countries.

## Results

### Wheat production and yield vis-à-vis climate trends

**Figure 1:**
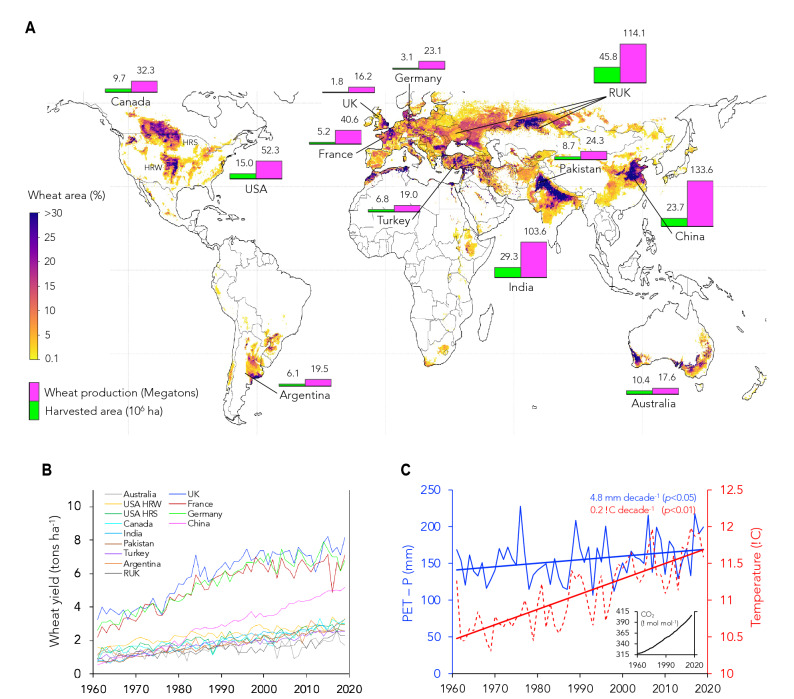
Global wheat area and trends in wheat yield and climate in top-twelve global wheat producers (1961–2019). (**A**) Worldwide wheat cropping area (%)^29^, total harvested area (10^6^ hectares in 2019), and wheat production (megatons for 2019) of the top 12 global wheat producers (China, RUK—Russia, Ukraine, and Kazakhstan, India, USA—hard red winter (HRW) and hard red spring (HRS), France, Canada, Pakistan, Germany, Argentina, Turkey, Australia, and United Kingdom) (Map was generated in Python 3.8.5; http://www.python.org). (**B**) Changes in wheat yield (tons per hectare) and (**C**) climate—mean daily temperature (red dashed line; °C) and the seasonal water balance represented as potential evaporation minus precipitation (blue line; PET—P in millimeters of H_2_O). A positive trend in PET-P indicates an increase in water deficit. The seasonal atmospheric [CO_2_] in μmol CO_2_ per mol^−1^ air is also shown in the insert of **C** (black line). Temperature, PET-P, and [CO_2_] shown in **C** are averaged values over the wheat-growing period and the shared area of the wheat-growing areas of the top 12 global wheat producers. Decadal trends in temperature (red) and PET-P (blue) as well as the significance levels of these trends are presented in **C**.

Wheat is currently grown in all six continents except Antarctica. The leading producers include China, the Russian Federation, Ukraine, Kazakhstan (RUK), India, USA, France, Canada, Pakistan, Germany, Argentina, Turkey, Australia, and United Kingdom (Fig. 1 and Supplementary Table 1). The total grain production of these twelve countries is estimated at 600 megatons (2019 data), which accounts for over 78% of the global wheat production. The top three producers are China with 133.6 megatons per year (Mt y^−1^), RUK with 114.1 Mt y^−1^, and India with 103.6 Mt y^−1^. RUK contains the largest harvested area of 45.8 million hectares, followed by India with 29.3 million hectares and China with 23.7 million hectares (Fig. 1A). Despite a relatively small harvested area of 10.1 million hectares (only 22% of RUK’s harvested area), the United Kingdom, France, and Germany account for the world’s highest yields per hectare, with 8.93 tons ha^−1^, 7.74 tons ha^−1^, and 7.40 tons ha^−1^, respectively (compared with the world’s average yield of only 3.2 tons ha^−1^), accounting for a total yearly production of 79.9 Mt y^−1^.

While all these twelve major wheat producers saw an increase in yield during the last six decades (Fig. 1B), China displayed the most noteworthy increase with a nearly sevenfold higher yield in 2019 than in 1961 and a mean total increase of 5.19 tons ha^−1^ for the period of 1961–2019. Germany, the UK, and France reported comparable yield increases of 5.20 tons ha^−1^, 5.19 tons ha^−1^, and 4.81 tons ha^−1^, respectively, during this period, suggesting an approximately 1.6-fold improvement since 1961 (Fig. 1B). Australia, RUK, and Turkey reported the lowest gains with only 0.87 tons ha^−1^, 1.26 tons ha^−1^, and 1.71 tons ha^−1^, respectively, representing improvements of 67%, 150%, and 175% in yield per hectare since 1961.

Yield increase occurred despite the steep rise in temperature (nearly 1.2 °C) in the twelve countries during the last six decades (Fig. 1C). Water deficit—calculated as the difference between potential evaporative demand and precipitation (PET—P; mm H_2_O y^−1^)—also increased by an average of $\sim$ 29 mm of H_2_O for the same period. Increases in yield since the early 1960s were likely due to breeding and agrotechnological advances, improved management, and a steep rise in atmospheric [CO_2_] of $\sim$ 98 μmol mol^−1^, from 315.9 μmol mol^−1^ in 1961 to 413.4 μmol mol^−1^ in 2019 (insert in Fig. 1C).

### Unraveling the impacts of climate and [CO_2_] on yield

Based on previous studies^30,31^, we used a log-linear model to quantify the impact of [CO_2_] and daily minimum (*T*_min_), maximum (*T*_max_), and mean (*T*_mean_) temperatures, as well as seasonal water deficit (PET-P), and rainfall distribution on wheat yield. Climate variables were obtained from the TerraClimate data set^32^, while monthly records of [CO_2_] from the Mauna Loa station were used to model the effects of CO_2_ (see “Methods”). To quantify wheat yield as a function of climate variables and [CO_2_], we included all 12 countries in the regression analysis. Supplementary Table 2 presents summary statistics of all variables, while Supplementary Fig. 1 depicts trends in *T*_mean_ and PET-P per country.

Since climate variables tend to be correlated over time (Supplementary Table 3), controlling for all of these variables in the model facilitates the estimation of their distinct effect on yield. We used country-specific trends to distinguish changes in wheat yield related to climate and [CO_2_] from those attributed to agrotechnological advancements, changes in country-specific policies, and other local-changing factors (e.g., economic and population growth; more information on how this was done can be found in “Methods”). We also included country-specific effects across all models to account for unobserved time-invariant heterogeneity at the country level, such as geographical properties, edaphic characteristics, and other local-specific features (see “Methods”).

Table 1 reports the estimated regression coefficients of four models, (1) using only temperature variables (T), (2) temperature and water-related (i.e., seasonal rainfall distribution and water deficit as PET-P) variables (T + W), (3) including [CO_2_] (T + W + C), and (4) the interaction between [CO_2_] and climate variables (T + W + C + interactions).

**Table 1:** Effects of climate variables and [CO_2_] on log wheat yields of the world’s major wheat producers.

Independent variable: Log(Yield)	T	T + W	T + W + C	T + W + C + interactions
*T*_mean_	− 0.0099***	− 0.0054*	− 0.010***	− 0.010***
	(0.0029)	(0.0034)	(0.004)	(0.004)
*T*_max_	0.00012	− 0.00008	0.00027	0.0003
	(0.00054)	(0.00056)	(0.00056)	(0.0006)
*T*_min_	0.0020	− 0.0002	0.0018	0.0016
	(0.0020)	(0.0022)	(0.0023)	(0.0023)
Rain distribution		− 0.0018	− 0.00166	− 0.0022
		(0.0088)	(0.0088)	(0.0088)
Water deficit (PET-P)		− 0.0004*	− 0.0006***	− 0.0007***
		(0.0002)	(0.00017)	(0.0002)
[CO_2_]			0.2503***	0.2684***
			(0.074)	(0.0756)
*T*_mean_ $\times$ [CO_2_]				− 0.114*
				(0.067)
*T*_max_ $\times$ [CO_2_]				0.0109
				(0.0162)
*T*_min_ $\times$ [CO_2_]				0.0994
				(0.0613)
Rain distribution $\times$ [CO_2_]				0.0636
				(0.2576)
Water deficit (PET—P) $\times$ [CO_2_]				0.0008*
				(0.0004)
Adj. Rsq	0.9668	0.9670	0.9675	0.9678
RMSE	0.0465	0.0463	0.0460	0.0461
N	767	767	767	767

From left to right column—T is the model with temperature variables only, T + W includes the water variables (rain distribution and water deficit as PET—P), T + W + C includes [CO_2_], and the T + W + C + interaction model includes the combined effect of [CO_2_] and climate variables. Standard errors are shown in parenthesis. Dependent variable is the logarithm of wheat yield. Rainfall distribution and [CO_2_] in the models are the logarithm of the coefficient of variation in seasonal rain (standard deviation divided by the monthly mean rainfall) and the logarithm of the mean atmospheric CO_2_ concentration (μmol mol^−1^) during the growing period. Stars indicate statistical significance: *p $\le$ 0.1, **p $\le$ 0.05, ***p $\le$ 0.01.

Among the temperature measures, only *T*_mean_ had a consistently significant effect on yield (*p* < 0.01, two-sided *t*-test), with increases in *T*_mean_ during the growing season leading to reduced wheat yields (negative effect) in all four models. This negative effect weakened (*p* < 0.1, two-sided *t*-test) when the water deficit variable was included in the model (T + W) but strengthened again (*p* < 0.01, two-sided *t*-test) when [CO_2_] was further incorporated (T + W + C and T + W + C + interactions models). The negative effect of water deficit suggests a reduction in annual yield when the evaporation demand is greater than precipitation because of reduced rainfall or increased evaporative demand. Rainfall distribution is an important variable in predicting wheat yields^30,33^. In our models, rainfall distribution had a negative effect on yield (Table 1), implying reduced annual yield when rainfall is less uniformly distributed throughout the season, consistent with other studies^30^. This effect is likely because of decreased water retention in the soil when rainfall is more intense and less frequent during the season. Notably, the negative effect of rainfall distribution in all models was not statistically significant (*p* > 0.1, two-sided *t*-test), indicating that rainfall distribution might be an important predictor of yield in some but not all places.

Among all variables, atmospheric [CO_2_] had the most substantial effect on yield, with a net beneficial effect in both T + W + C and T + W + C + interaction models. This effect was two and four orders of magnitude higher than T and W, respectively (Table 1). This much stronger effect of [CO_2_] indicates that the [CO_2_] $\times$ T or W interaction is mainly through T and W moderating or enhancing the effect of [CO_2_] and not the other way around. The negative sign of the *T*_mean_ $\times$ [CO_2_] interaction (Table 1) indicates that the adverse (negative) impact of warming on yield weakens under elevated [CO_2_] (i.e., when atmospheric [CO_2_] rises), consistent with previous experimental studies^34^. Interestingly, the water deficit $\times$ [CO_2_] interaction was positive suggesting that a rise in [CO_2_] increases the negative effect of water deficit on yield, contrary to prior studies^12,13,21,35^.

Since the irrigated area of wheat in India increased dramatically from a low 10% in the early 1970s to almost 100% by the late 2000s with a substantial impact on wheat yield^36^, we further repeated the analysis by excluding India from our models. As shown in Supplementary Table 4, the estimated regression coefficients of the four models that excluded India from the analysis are similar to those presented in Table 1 when India was included in the model. The only exception was the insignificant negative *T*_mean_ $\times$ [CO_2_] interaction (*p* > 0.1) when India was excluded from the analysis (Supplementary Table 4), which implies that a significant proportion of the beneficial effect of [CO_2_] that neutralized the negative impact of warming was due to the increased irrigation in India^36^.

### Yield changes due to climate and [CO_2_]

We further analyzed yield changes due to climatic variations and [CO_2_] rise by applying regression analysis under different scenarios adjusting climate variables and [CO_2_] to the levels reported in 1961–1965. We then established the T + W + C model (Table 1) with each variable fixed at its 1961–1965 average, each variable at a time. We translated these effects into relative changes in yield due to climate and [CO_2_] by calculating the difference between predicted yields using our baseline regression and predicted yields using climate variables or [CO_2_] levels recorded in 1961–1965. We also assessed current changes for each country separately by estimating the contribution of [CO_2_] and each of the climate variables to the yield in 2018–2019 compared with the baseline yield in 1961–1965.

**Figure 2:**
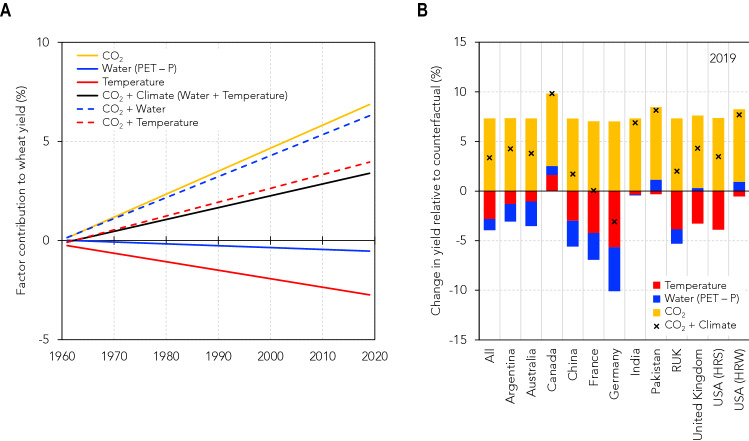
Climate and [CO_2_] contributions to variations in wheat yield. (**A**) Percent change in wheat yield calculated as the relative difference between the original regression model and the model with values for temperature (red solid line), water deficit (blue solid line), [CO_2_] (yellow solid line), and all three combined (temperature, water deficit, and [CO_2_]; black solid line) fixed at 1961–1965 levels. The graph is presented as the relative contribution of each of the variables to the change in wheat yield. The contribution of the combinations: temperature + [CO_2_] and water deficit + [CO_2_] are also presented as red and blue dashed lines, respectively. (**B**) The separate contribution of temperature (red bars), water deficit (blue bars), and [CO_2_] (yellow bars) to the change in wheat yield in 2018–19 compared with the baseline of 1961–1965 for each of the top 12 global wheat producers. The total contribution of both climate and [CO_2_] is noted with an “x” symbol.

In general, the benefits of [CO_2_] rise outweighed the adverse impact of warming and water deficit over time (black line in Fig. 2A). The yield gained due to an almost 100 μmol mol^−1^ increase in [CO_2_] was 6.8% on average, with a linear increase from 1961 to date (yellow line in Fig. 2A). Such an increase in wheat yield per $\Delta$[CO_2_] of $\sim$ 100 μmol mol^−1^ is within the range of 5–10% reported from FACE experiments for a similar increase in [CO_2_]^12,13^. Warming of $\sim$ 1.2 °C from 1961 to 2019 (Fig. 1C) reduced the annual yield by nearly 2.8% (red line in Fig. 2A), and an increased water deficit of 28.8 mm H_2_O m^–2^ for the same period reduced the yield by almost 1% (blue line in Fig. 2A). On average, the gain in wheat yield from the recent rise in atmospheric [CO_2_] was enough to overcome the loss due to warming and water shortage (red and blue dashed lines in Fig. 2A), consistent with other studies^37^. The finding that warming of $\sim$ 1.2 °C suppressed the yield by $\sim$ 3% is slightly lower but generally in line with previous grid-based and point-based simulations^8,9^. However, despite the previously reported benefit of elevated [CO_2_] on yield under drought conditions^20,38^, our findings of a positive water deficit $\times$ [CO_2_] interaction (Table 1 and Fig. 2A) and reduced yields in the presence of increased [CO_2_] combined with intense drought (blue dashed line in Fig. 2A and Supplementary Fig. 1) question this conclusion.

Figure 2B presents the relative change in yield across the 12 major wheat-producing countries in 2018–19. Yield gains due to the beneficial effect of [CO_2_] (yellow bars in Fig. 2B) outweighed losses due to warming and intensified drought in most countries (red and blue bars in Fig. 2B). Germany and France—two of the three countries with the highest yield per hectare in the world (Fig. 1B)—were the only countries that saw loss (− 10.1% due to warming and drought compared to + 7% due to [CO_2_] rise) and no gain (− 7% due to warming and drought, and + 7% due to [CO_2_] rise) in yield, respectively, at the end of the sampling period relative to the 1961–1965 baseline. These two countries experienced the worst temperature rise and intense drought conditions in their wheat-growing areas at the end of the 2010s (Supplementary Fig. 1), enough to obliterate or reverse the gains due to the rise in atmospheric [CO_2_] into significant yield losses. The largest wheat producers—China and RUK—also experienced a substantial decrease in yield, with losses of 5.6% and 5.3%, respectively, mainly due to similar warming of + 0.31 °C decade^–1^ (*p* < 0.01) in both regions, and a water deficit of 21.5 mm decade^–1^ (*p* < 0.05) in China and 6.1 mm decade^–1^ (*p* < 0.1) in RUK (Supplementary Fig. 1). Nevertheless, such losses were insufficient to reverse gains in these two countries due to the rising [CO_2_] in the last six decades (Fig. 2B).

## Discussion

Apart from the consistent rise in temperature and atmospheric [CO_2_] during the last six decades, drought has become more frequent and intense, not only in dry areas but also in places where drought rarely occurred before^1^. Such was the case for a wheat-growing area of $\sim$ 1.66 million km^2^ (nearly the size of Mexico), an area that corresponds to over 78% of the global wheat harvested area, during the last six decades. Both warming and drought-induced water deficits were significant in this area, negatively affecting yields. Climatic changes triggered large year-to-year and spatial variability in yield, with up to 45% of the variation occurring in highly productive countries such as the United States, Canada, the United Kingdom, Turkey, Australia, and Argentina^7^. A temperature increase of 1 °C was associated with a decline in wheat yield between 4.1% and 6.4% by three independent methods^8^. Our results based on census data are more conservative, showing a yield  reduction of $\sim$ 2.4 $\pm$ 1.2% per 1 °C, consistent with a previous metanalysis^37^. Morever, including the warm year of 1961 in the baseline might have resulted in a slight underestimation of the temperature effect in our analysis. Our analysis also shows that the mean temperature is the only temperature measure affecting yield. In contrast, temperature extremes (i.e., maximum and minimum) had no significant effect on the yield (Table 1). This lack of effect can be attributed to the temperature pattern in the last six decades, which was much more moderate than today. However, as climate change is expected to increase the frequency and magnitude of extreme weather events (e.g., heatwaves), the effect of minimum/maximum temperatures on yield is likely to become more significant in the future^39^.

Most of the studies that analyze changes in regional wheat yield due to climate variations use process-based crop models^40^. These models usually do not account for [CO_2_] effects, likely underestimating future yields^28^. Models that do include [CO_2_] assume a positive, usually linear, or near-linear effect of [CO_2_] under both warming and drought conditions (e.g., Ref^27^), which might result in an overestimation of yield under future climate scenarios according to our analysis. Models that include [CO_2_] effects rely on equations derived from experimental observations showing average reductions in stomatal conductance of $\sim$ 21%^18^ and evapotranspiration of $\sim$ 10% for all investigated C3 crops under elevated [CO_2_] levels of + 100 μmol mol^−1^ to + 150 μmol mol^−1^^13^. These reductions suggest that water-use efficiency will improve under a future increase in [CO_2_]^20^ increasing the drought tolerance of C3 crops, like wheat. Though CO_2_ has been shown to offset the negative effect of heat stress^34^, our results indicate that the interaction might be more complex under extreme drought conditions than previously suggested.

A recent study involving five FACE facilities in Germany, Italy, China, Australia, and the USA under relatively dry conditions with total rainfall during the growing season ranging between 141 and 347 mm year^–1^ showed that exposure to elevated [CO_2_] of + 150 μmol mol^−1^ did not affect yield and photosynthetic rate of wheat in almost all sites (with the only exception of the USA site)^41^. In general, the lack of a CO_2_ effect on yield was explained by an unchanged assimilation rate, concluding that the effect of CO_2_ on yield is highly dependent on local environmental conditions (e.g., water availability, N fertilization rate, soil type), acclimation capacity, and cultivar. An eight-year FACE experiment in a dry environment showed significant variation in soybean (C3 crop) yield under elevated [CO_2_]. These variations were attributed to the amount of water in the soil available to the crop throughout the growth period^23^. In contrast to previous expectations^21^, it was found that high [CO_2_] did not necessarily conserve water in the soil via decreased stomatal conductance but may have even caused losses in some years, with water losses leading to lower yields. Surprisingly, the effect of [CO_2_] on soil water was not correlated with its effect on midday stomatal conductance (see Fig. 3b in Ref^23^). A greater canopy temperature due to reduced cooling via lower transpiration and a greater leaf area index (the one-sided area of all leaves in a square meter of the ground) were both suggested to cause the unexpectedly greater water losses observed in drought years. These negative water and energy balances offset the beneficial effect of [CO_2_] on stomatal conductance, leading to net losses in soil water content and, consequently, grain yield.

These results, as well as those from our study, suggest that conclusions from previous FACE experiments should be carefully re-examined in light of local growing conditions and cultivars used in the experiments. Variations in the results of FACE experiments conducted at different locations pose a great challenge to the study of crop response mechanisms to elevated [CO_2_] under climate scenarios. In fact, FACE experiments conducted in different locations may not be comparable due to differences in soil and climate conditions and because of a different response of the cultivars, usually bred under different climates. Since FACE experiments are expensive and thus rarely performed, results from the few FACE facilities are usually extrapolated to unrepresented areas. However, extrapolating FACE results to currently dry and warm regions like Africa and the Middle East, for example, might be a challenge in the absence of prior FACE experiments in such regions. The inclusion of FACE as well as other long-term [CO_2_] enrichment experiments in these regions that are already experiencing severe warming and drought (e.g., many parts of Africa and the Middle East) may enhance our understanding of crop response to elevated [CO_2_] in cold and humid areas susceptible to future drier and warmer conditions and potential shift towards dryland-controlled mechanisms^42^. Studying the response of different lines bred under warm and dry conditions is also recommended to analyze crop breeding adaptation to altered environmental and climate conditions^43^.

An obvious next step should be the use of biophysical simulation models to compare their attribution to the present statistical analysis. We expect this call to precipitate such participation and analysis from various groups worldwide dealing with the study of climate and [CO_2_] impacts on crops. Comparing these results with model simulations or long-term fixed experiments data could deepen our understanding of the underlying mechanisms of crop response to climate and future elevated [CO_2_] at the regional and global scales. Moreover, six decades of census observation data may be leveraged to examine simulation model performance under actual [CO_2_] rise of almost 100 μmol mol^−1^. The combination of statistical analysis, like the one presented here, and simulation modeling or long-term fixed experiments data could also shed light on the various roles of agrotechnology, local environment, and cultivar in affecting wheat production, with important implications for climate change adaptation efforts. To notice is that our model currently assumes a linear rise of [CO_2_] and would require an adjustment to a non-linear increase in [CO_2_] to predict wheat response under future emission scenarios.

Finally, since the response of C3 plants to [CO_2_] is non-linear, the beneficial effect of [CO_2_] on photosynthesis reaches a steady state around $\sim$ 700–800 μmol mol^−1^^44^. These are the levels expected by 2100^1^. It is likely that [CO_2_] will no longer cushion the impact of warming and drought by the end of this century. An atmosphere with a doubled concentration of CO_2_ will no longer increase photosynthesis while corresponding warming between 1.0 and 3.7 °C, accompanied by more frequent and intense droughts, will likely have a detrimental effect on global food security. Further, a substantial body of work suggests that a CO_2_-rich atmosphere decreases food quality by reducing protein and mineral contents^45^, with alarming consequences for human nutrition. Establishing a commonly-shared protocol for FACE-like experiments and expanding FACE studies to warmer and drier areas such as Africa and the Middle East is essential to increase our understanding of the impact of climatic and environmental conditions on crop yield and quality for appropriate adaptation and mitigation strategies.

## Methods

### Data

We collected census data on wheat yields for the top 12 global wheat-producing countries (China, RUK—Russia, Ukraine, and Kazakhstan, India, USA—hard red winter (HRW) and hard red spring (HRS) wheat areas, France, Canada, Pakistan, Germany, Argentina, Turkey, Australia, and United Kingdom) for the period 1961–2019 from the Food and Agriculture Organization (FAO; http://faostat.fao.org). These census data sets include information related to the total harvested area in hectares for each country and specific year, as well as the total yearly seed production in tons. Data for the wheat-producing areas of the former Soviet Union is provided until 1991. Thus, we had to complete the data for these areas for 1992–2019 by summing the harvested areas and wheat seed productions of the Russian Federation, Ukraine, and Kazakhstan (RUK), which represent 75% of the former Soviet Union wheat-producing areas.

Gridded monthly climate data at 2.5 arc minutes ($\sim$ 1.85 km $\times$ $\sim$ 1.85 km at the equator) for the period 1961–2019 were obtained from TerraClimate: Monthly Climate and Climatic Water Balance for Global Terrestrial Surfaces data set of the University of Idaho^32^. Spatially-weighted averages of gridded precipitation (P), maximum and minimum temperatures (*T*_max_, *T*_min_), and potential evapotranspiration (PET) were computed for each growing season based on weights defined by the wheat cropped area from Ray et al. (2012). An averaged area over 1995–2005 wheat cropped area maps was used to represent the mean harvested area for each country during the last six decades since the change in the spatial extent of the harvested area from this period was relatively small (Supplementary Fig. 3). The mean temperature (*T*_mean_) was calculated as the average of *T*_max_ and *T*_min_. Rainfall distribution was calculated as the coefficient of variation in rainfall (i.e., *P*_*CV*_, calculated as the standard deviation divided by the mean) along the growing season using monthly rainfall levels. A large *P*_*CV*_ means that most of the rainfall was concentrated in a specific month (or a few months) along the season, while a small *P*_*CV*_ means a more evenly distributed rainfall across the season. Monthly [CO_2_] data collected from the Mauna Loa station since 1958 were downloaded from NOAA Global Monitoring Laboratory website (https://gml.noaa.gov/ccgg/trends/data.html) and averaged over the duration of the country-specific growing season period. Growing seasons are determined according to the reported sowing, maturity, and harvest dates of wheat in each country or country-specific region (e.g., HRW and HRS wheat-growing areas in the USA) (Supplementary Table 1).

We used PET-P as a proxy of water deficit conditions. PET-P is more representative of water deficit conditions than P because it reflects the balance between evaporative demand and water supply by rainfall. Though PET-P does not include water supply by irrigation, most of the wheat-growing areas in the world are rainfed, suggesting that the limited amount supplied by irrigation has a minor effect on yields in the model. This is less true, however, for India in which a meaningful proportion of its cropped area is intensively irrigated. Moreover, India saw an increase in its irrigated area from a low 10% irrigated area in the early 1970s to almost full coverage in the 2000s, with a substantial effect on the yield^36^. Repeating the analysis and excluding India from the models, however, did not alter the results except for the *T*_mean_ $\times$ [CO_2_] interaction (Table 1), which was no longer significant when India was excluded from the analysis (Supplementary Table 4).

### Assessing the impact of climate and [CO_2_] on yield

We used a well-established methodology to predict the logarithm of wheat seed yield based on climatic and environmental factors using a multivariate log-linear regression ^30,36^. Four models were tested: (1) using only temperature variables (T), (2) temperature and water-related (i.e., seasonal rainfall distribution and water deficit as PET-P) variables (T + W), (3) including [CO_2_] (T + W + C), and (4) the interaction between [CO_2_] and climate variables (T + W + C + interactions). The complete log-linear regression model is as follows:

1 $$log\left\{ {{\text{Y}}_{i,t} } \right\} \, = \alpha_{1} \cdot T_{{{\text{min}},i,t}} + \alpha_{2} \cdot T_{{{\text{max}},i,t}} + \alpha_{3} \cdot T_{{{\text{mean}},i,t}} + \alpha_{4} \cdot P_{CV,i,t} + \alpha_{5} \cdot \left\{ {{\text{PET}} - {\text{P}}} \right\}_{i,t} + \beta_{1} \cdot T_{{{\text{min}},i,t}} \cdot log\left\{ {\left[ {{\text{CO}}_{{2}} } \right]_{i,t} } \right\} \, + \beta_{2} \cdot T_{{{\text{max}},i,t}} \cdot log\left\{ {\left[ {{\text{CO}}_{{2}} } \right]_{i,t} } \right\} \, + \beta_{3} \cdot T_{{{\text{mean}},i,t}} \cdot log\left\{ {\left[ {{\text{CO}}_{{2}} } \right]_{i,t} } \right\} \, + \delta_{1} \cdot P_{CV,i,t} \cdot log\left\{ {\left[ {{\text{CO}}_{{2}} } \right]_{i,t} } \right\} \, + \delta_{2} \cdot \left\{ {{\text{PET}} - {\text{P}}} \right\}_{i,t} \cdot log\left\{ {\left[ {{\text{CO}}_{{2}} } \right]_{i,t} } \right\} \, + \gamma_{1} \cdot log\left\{ {\left[ {{\text{CO}}_{{2}} } \right]_{i,t} } \right\} \, + \theta_{i,t} + C_{i} + \epsilon_{i}$$

where Y_*i,t*_ and [CO_2_]_*i,t*_ denote wheat seed yield (in tons ha^–1^) and mean seasonal [CO_2_] (in μmol mol^−1^) in location (country) *i* and year *t*. Transformation to log yields and log [CO_2_] results in normally distributed values and highly proportionate to the effects of climate variables and [CO_2_] on yield (A linear yield model was also tested with a similar results and conclusions pattern; Supplementary Table 5). *T*_min,*i,t*_, *T*_max,*i,t*_, and *T*_mean,*i,t*_ denote the minimum, maximum and mean temperatures, respectively, during the growing season in location *i* and year *t*, while *P*_*CV,i,t*_ and {PET-P}_*i,t*_ are the water-related variables, i.e., the coefficient of variation in seasonal rainfall and the water deficit (in mm H_2_O y^–1^) calculated as the balance between evaporative demand (PET) and water supply as rainfall (P)—in location *i* and year *t*, respectively.

Based on previous statistical studies of changes in yield due to climate, we included country-specific time-invariant controls (*C*_*i*_), as well as country-specific trends ($\theta$_*i,t*_). Accounting for these controls is necessary to isolate potential influences of exogenous changes in the climate variables on wheat yields. For $\theta$_*i,t*_, we used a quadratic time trend in yields for each specific country^30^.

The $\alpha$ coefficients in Eq. (1) represent the magnitude of the effect of the climate parameters (temperature and water deficit) on yield, while ${\gamma }_{1}$ represents the effect of [CO_2_]. The ${\varvec{\beta}}$’s and ${\varvec{\delta}}$’s in Eq. (1) are the interaction coefficients, whereas ${{\varvec{\beta}}}_{1},$ ${{\varvec{\beta}}}_{2}$, ${{\varvec{\beta}}}_{3}$ represent a measure of the effect of temperature variables on the sensitivity of wheat yield to [CO_2_], and ${{\varvec{\delta}}}_{1}$ and ${{\varvec{\delta}}}_{2}$ denote similar parameters for rainfall distribution and water deficit, respectively.

Thus, for models 1–3 (T, T + W, and T + W + C models) ${\varvec{\beta}}$ and ${\varvec{\delta}}$ coefficients are all equal to zero while in model 1 (T model) ${\alpha }_{4}$ = ${\alpha }_{5}$ = ${\gamma }_{1}$  = 0 and in model 2 (T + W model) ${\gamma }_{1}$ = 0.

### Contribution of temperature, water deficit, and [CO_2_] to changes in yield

We used the estimates derived from the T + W + C statistical model to assess the contribution of climate variables (temperature and water deficit) and [CO_2_] to changes in yield over time. We do so by calculating the percent change between predicted yields from our main regression model and predicted yields from a counterfactual scenario using the years 1961–1965 as the baseline. Though the year 1961 was exceptionally warm in some countries (see Supplementary Fig. 1), we decided to include it in the baseline because it represents the abnormal variability in global temperature conditions since industrial times. We used four counterfactual scenarios: (1) maintaining [CO_2_] at the 1961–1965 average level, (2) holding temperature variables (*T*_min_, *T*_max_, and *T*_mean_), and (3) water deficit (PET-P) at the 1961–1965 average level, and (4) maintaining all climate variables (temperature and water deficit) as well as [CO_2_] at the 1961–1965 average level, to measure the individual and overall impacts of climate and [CO_2_] on the yield of each major wheat producer. We added the countrywide percent changes to determine the total impact of climate and [CO_2_] on yields in the shared cropped area of the top 12 global wheat-producing countries.

### Caveats

There are various caveats associated with our analysis. Since we used a multivariant regression model, we were unable to determine the indirect effect of [CO_2_] on yield via its effect on temperature and water deficit. Also, our model assumes linear relationships, while some of the interactions might be non-linear in nature. Our models include country-specific effects and country-quadrate trends to predict yields under the counterfactual scenarios. We controlled for such effects assuming that they were independent of climate and [CO_2_] trends specifically, and were mostly related to agrotechnological advances, changes in country-specific policies, and other local-changing factors such as economic and population growth^30^. While this is likely to be true for most cases, minor effects of climate and [CO_2_] might have been eliminated thereby resulting in dependence on model specifications. Also, significant yield losses may be caused by weather extremes at short periods of days and even hours when the wheat reaches a crucial developmental stage while our analysis is confined to average seasonal conditions. Warming is expected to shorten the growth season (rapid phenology)^46^ and decrease the yield production potential as crops absorb less radiation. However, it can be expected that breeders can overcome this through late phenology breeding. [CO_2_] is uniformly distributed across the globe, and thus we used a single-point measurement to represent [CO_2_] levels in the different wheat-producing countries. Despite seasonal variations in [CO_2_] among the countries because of differences in the growing season periods, such variations are relatively small and likely underestimate the real effect of [CO_2_] on yield in some places.

## Supplementary Information

Supplementary Information.
