# Supplementary material for: Six decades of warming and drought in the world’s top wheat-producing countries offset the benefits of rising CO2 to yield
Source: Sci Rep. 2022 May 13;12:7921. doi: 10.1038/s41598-022-11423-1 (PMC9106749; doi:10.1038/s41598-022-11423-1)
Supplement: Supplementary file 1 — Supplementary Information. [file 41598_2022_11423_MOESM1_ESM.docx]

**Supplementary Information for**

**Six decades of warming and drought in the world’s top wheat-producing countries offset the benefits of rising CO_2_ to yield**

David Helman^1,2,*^ and David J. Bonfil^3^

1. Institute of Environmental Sciences (Soil & Water Sciences), The Robert H. Smith Faculty of Agriculture, Food and Environment, The Hebrew University, Rehovot 7610001, Israel
2. Advanced School for Environmental Sciences, The Hebrew University, Jerusalem, Israel
3. Department of Vegetable and Field Crop Research, Agricultural Research Organization, Gilat Research Center, 8531100, Israel

^*^ Corresponding author: David.helman@mail.huji.ac.il

**Supplementary Information**

**Supplementary Note 1**

**Data Sources**

TerraClimate is a monthly climate and climatic water balance data set for global terrestrial surfaces^1^. It uses climatically aided interpolation through combining high-spatial-resolution climatological normals from the WorldClim dataset and coarser spatial resolution but time-varying data from CRU Ts4.0 and the Japanese 55-year Reanalysis (JRA55). Conceptually, the procedure applies interpolated time-varying anomalies from CRU Ts4.0/JRA55 to the high-spatial-resolution climatology of WorldClim to create a high-spatial-resolution dataset that covers a broader temporal record.

Temporal information is inherited from CRU Ts4.0 for most global land surfaces for temperature, precipitation, and vapor pressure. However, JRA55 data is used for regions where CRU data had zero climate stations contributing (including all of Antarctica, parts of Africa, South America, and scattered islands). For primary climate variables of temperature, vapor pressure, and precipitation, the University of Idaho provides additional data on the number of stations (between 0 and 8) that contributed to the CRU Ts4.0 data used by TerraClimate. JRA55 was used exclusively for solar radiation and wind speeds.

TerraClimate additionally produces monthly surface water balance datasets using a water balance model that incorporates reference evapotranspiration, precipitation, temperature, and interpolated plant extractable soil water capacity. A modified Thornthwaite-Mather climatic water-balance model and extractable soil water storage capacity data is used in TerraClimate at a 0.5° grid^1^.

*A few limitations of TerraClimate:*

TerraClimate will not capture temporal variability at finer scales than parent datasets and thus is not able to capture variability in orographic precipitation ratios and inversions. Also, the water balance model is very simple and does not account for heterogeneity in vegetation types or their physiological response to changing environmental conditions.

**Methods**

We used in our analysis census data of wheat yields of the top-twelve wheat-producing countries: China, India, USA, France, Canada, Pakistan, Germany, Argentina, Turkey, Australia, United Kingdom, and the main areas in the former Soviet Union (Fig. 1 and Supplementary Table 1). To complete the data for the wheat areas of the former Soviet Union region after 1991, we summed the yearly shared harvested areas and yield productions of three countries, the Russian Federation, Ukraine, and Kazakhstan (RUK), from 1992 to 2019. The total wheat area of these three countries together comprises more than 75% of the harvested area of the former Soviet Union region. Additionally, as hard red winter (HRW) and hard red spring (HRS) wheat are grown in different periods (Supplementary Table 1) and across different areas in the USA, we treated these two areas separately, with annual temperatures and water deficits being estimated for each area in separate.

The relative importance of wheat (compared to other crops) per country is presented in Supplementary Figures 2 and 3. Following is a short description of the main wheat-growing characteristics per country and a reference to whether a field experiment with enriched [CO_2_] has been ever conducted on wheat in the studied country. The main features, including cropping cycle months, harvest area, and yield production, are summarized in Supplementary Table 1.

*Argentina*

Argentina is a major wheat exporter, with Brazil being the main importer of wheat from Argentina. The sowing date is in June with harvesting usually ending in January (Supplementary Table 1). While the wheat area in Argentina was 6.1 million hectares in 2019/2020, it is expected to increase to ​​6.5 million hectares by 2022/23. No CO_2_ enrichment field experiments have been conducted in Argentina. Thus, the response of local wheat cultivars to elevated [CO_2_], especially under warming and drought, is currently unknown for this area.

*Australia*

Wheat is the major winter crop grown in Australia with sowing starting in autumn and harvesting usually occurring in spring and summer (Supplementary Table 1). The main producing states are Western Australia, New South Wales, South Australia, Victoria, and Queensland. Various different types of wheat are being produced in Australia, including Australian Prime Hard (APH), Australian Hard (AH), Australian Premium White (APW), Australian Noodle Wheat (ANW), Australian Standard White (ASW), Australian Premium Durum (ADR), and Australian Soft (ASFT).

Two FACE facilities – at Horsham and Walpeup, Victoria – were operating in Australia from 2007 to 2018 (AGFACE)^2^. It was the first and only long-term FACE experiment (>10 years) established in a region with such warm and dry conditions. Many important results and conclusions were drawn from the Australian AGFACE (e.g., ^3,4^), including assessment of the effects of heatwaves on wheat and the prediction of yield under climate scenarios using numerical models, which were directly evaluated with the data collected from AGFACE ^5^. The main results indicated an increase of wheat yield of 12%-56%, depending on the site and treatment, for [CO_2_] of 550 μmol mol^−1^. It was claimed that elevated [CO_2_] was capable of buffering for the negative impact of heatwaves for Australian cultivars^4^.

*Canada*

Wheat is the most important crop grown in Canada, competing only with canola, with spring wheat comprising 72% of the total cultivated wheat. There are several classes of common wheat, based on factors including seed hardness and color, sowing time (autumn or spring), and the region where the varieties are grown. Nearly half of all Canadian wheat is grown in Saskatchewan, Alberta, and Manitoba. Canadian wheat ripens between the end of August and early September. Though the yield per hectare in Canada is within the global average (only 3.2 tons ha^–1^), farms are large enough to make large profits. No [CO_2_] enrichment field experiments were ever conducted in Canada.

*China*

As for 2019/2020, China is the top country in wheat production globally, with 133.6 megatons per year (Fig. 1A). Wheat has been widely grown as a staple food in China for around 4500 years, mainly in the northern part of the country and towards the northeastern parts (Fig. 1A). Roughly two-thirds of the total wheat production comes from north China plains and nearly another third from the central provinces. Most of the wheat is winter wheat making up to 87% of the wheat sown area, with the remaining being spring wheat. Sowing months are September to May, while harvesting occurs from May to June (Supplementary Table 1). There were two FACE facilities in China that tested the effect of elevated [CO_2_] on wheat in the last two decades. The first FACE site was established in Yangzhou, Jiangsu, where an [CO_2_] increase of +200 ppm was tested on wheat, with a reported increase in yield of 15% during 2007-2008 ^6^. The second facility was established in Changshu, Jiangsu, where the field environment was enriched to 500 ppm, and warming was exerted on the wheat in 2013-2014 ^7^. The yield was reported to increase in this experiment by 6%-10%, compared to ambient [CO_2_] conditions.

*France*

France’s wheat production is concentrated mainly in the northern regions of the country but to some extent, is grown throughout the entire country. The department of Centre leads the production of wheat in France, producing 16% of France’s total wheat supply. France is one of the three leading countries with the highest yield per hectare and the top producing country in Europe with nearly 40 megatons per year (Supplementary Table 1 and Fig. 1B). With the UK and Germany, France has increased its yield enormously in the last six decades. Sowing occurs from October to June while harvested usually in July and August. Surprisingly, FACE experiments have never been conducted in France; thus, we currently lack information about how local cultivars in France will respond to future high [CO_2_], particularly under projected warming and more intensified droughts.

*Germany*

After UK and France, Germany is the largest wheat producer in Europe, with an average yield per hectare of 7.40 tons ha^-1^ recorded for 2019/2020. Bayern is the top federal state where wheat is being cultivated in Germany. As of 2016, wheat production in Bayern was 17.2% of Germany's total production. The other top 5 states are Lower Saxony, Saxony-Anhalt, Mecklenburg–West Pomerania, and North Rhine-Westphalia, which account for 64.3% of Germany’s wheat production. Sowing in Germany is from fall to summer with harvest occurring typically in August (Supplementary Table 1).

Two FACE facilities were established in Germany, which tested the effects of elevated [CO_2_] on wheat. The first FACE facility was established in Stuttgart, which examined the effect of elevated [CO_2_] of +150 ppm on wheat yield in 2004-2006 ^8^ and 2008 ^9^. The yield increase by of 1%-10% under elevated [CO_2_] compared to ambient [CO_2_] conditions. The second facility was established during 2014-2015 in Brunswick, in which the exposure of winter wheat to [CO_2_] of 550 ppm under various nitrogen treatments was examined. Yield was reported to increase by 12%-16% ^10^ under 550 ppm and by 9%-17% under 600 ppm ^11,12^.

*India*

Wheat is the primary cereal crop cultivated in India. Globally, India is the second-largest wheat producer after China, with an annual production of 104 megatons y^–1^. Wheat is grown in India, mainly in the northern states. Uttar Pradesh is the top-most contributor of wheat with a total production of 25.2 megatons per year, followed by Punjab (15.8 megatons per year) and Madhya Pradesh (14.2 megatons per year). To avoid the summer monsoon, wheat in India is grown during the dry season, which spans approximately from October through February (Supplementary Table 1). The irrigated wheat area in India increased dramatically since the early 1970s, reaching almost full coverage by the late 2000s. Such an increase in the irrigated area impacted wheat greatly by boosting increases in yields and productivity across India^13^. Only one FACE experiment has been conducted in India that tested the effect of elevated [CO_2_] on wheat. The facility established in New Delhi in 2010-2012 showed that wheat yield increases by 15% under elevated [CO_2_] of 550 ppm ^14^.

*Pakistan*

Wheat is the main staple food crop in Pakistan, dominating all crops in harvested area and production. It accounts for 37.1 % of the crop area, 65% of the food grain harvesting area, and 70% of crop production. Punjab province is the leading wheat-producing region in Pakistan, accounting for 75% of the national output. Pakistan produces around 24 megatons per year, being among the top-ten global producers of wheat. Like in India, wheat is grown in Pakistan during the dry season from October to March, with harvest time from April to June (Supplementary Table 1). There are no [CO_2_] enrichment field experiments on wheat reported in Pakistan.

*RUK (Russia, Ukraine, and Kazakhstan)*

The wheat-growing area across the region of the former Soviet Union is the largest in the world. It is one of the top-three world wheat-producing areas in terms of annual production. Since 1991, most of the wheat from this region come from areas within the Russian Federation, Ukraine, and Kazakhstan (RUK). In total, the wheat area of these three countries is the largest in the world, with nearly 46 million hectares of cultivated wheat. Yet, RUK has one of the lowest yields per hectare (Fig. 1B). Wheat is being cultivated in the Russian Federation across the majority of the country’s regions with Rostov being the main and the most productive (13% of total wheat harvest in Russia). In Ukraine, wheat is mainly grown in the central and south-central parts of the country; however, in general, it is being grown throughout the entire country. Kazakhstan is one of the world's major wheat and flour exporters, being among the ten largest producers. About 75% of Kazakhstan’s wheat is produced in three oblasts in north-central Kazakhstan: Kostanay, Akmola, and North Kazakhstan. We focused our study on winter wheat in RUK, which is the main wheat grown in this region. No FACE experiment has ever been conducted in this region. Thus, we currently lack any knowledge about the potential responses of wheat grown in these areas to elevated [CO_2_], especially under future climate.

*Turkey*

Wheat is cultivated all over Turkey, with the Central Anatolia region accounting for 57% of the Country’s total production. With a total harvested area of nearly 7 million hectares, Turkey produces around 19 megatons per year. The sowing period in Turkey is from September to June, with a harvest time around July-August (Supplementary Table 1). There have not been reports of [CO_2_] enrichment studies on wheat in Turkey.

*United Kingdom*

The UK currently produces the world’s highest wheat yield per hectare, with 8.93 tons ha^-1^. Wheat is a popular crop grown in the UK likely because of its twice a year sowing, during the autumn and spring, with harvest time in August. Milling wheat is the most popular in the UK. Most UK farmers opt to sow in autumn (October), allowing the crop to grow through winter, which tends to produce higher yields than the spring crop and allows farmers to spot and remove any early growing black grass. In general, the combination of the UK’s frequent showers and heavy soils provide agreeable conditions for wheat growth. Although June rainfall is critical for adding weight to filling grains, solar radiation during this month is likely the most important factor for yield formation. As in France, no [CO_2_] enrichment field experiments were ever conducted in the UK. Thus, responses of local wheat cultivars to elevated [CO_2_] are currently unknown.

*USA (HRS and HRW)*

Wheat ranks third among USA field crops in planted area, production, and gross farm receipts—behind corn and soybeans. It is cultivated across the entire USA. The two main types of wheat grown in the USA are the hard red spring (HRS) and hard red winter (HRW) wheat. About 95% of the HRS is grown in the states of North Dakota, Minnesota, Montana, and South Dakota while North Dakota accounts for slightly more than half of the annual HRS production in the USA. In contrast, HRW is grown in the Great Plains, Pacific Northwest (PNW), and California, and it is the most widely grown class in the United States. Because of the large growing area, conditions can significantly vary among the production regions with essential variations in yield and quality. With a total area of 52 million hectares, the production of HRS and HRW is around 52 megatons per year, accounting for a relatively low yield per hectare, especially compared to China and the leading European countries (Fig.1 and Supplementary Table 1).

The USA was the first to establish a FACE facility in the early 1990s’. The FACE experiment in Maricopa, Arizona, which tested the response of wheat to [CO_2_] of 550 ppm was the first to report the beneficial effect of elevated [CO_2_] under drought conditions, with increases up to 8%-20% ^15^. The following experiment in 1996-1997 under elevated [CO_2_] of +200 ppm and water and nitrogen limitations also showed increases in yields of 11%-14% ^16^.

**Supplementary Figures**

Supplementary Figure 1. (Left) Percent change in wheat yields calculated as the relative difference between the original T + W + C regression model and the T + W + C model with values for temperature (red solid line), water deficit (blue solid line), [CO_2_] (yellow solid line), and all three factors together (temperature, water deficit, and [CO_2_]; black solid line) fixed at 1961 levels for each of the twelve-leading wheat producers. The relative contribution of each of the variables to the change in wheat yields is presented while the contribution of the combinations: temperature + [CO_2_] and water deficit + [CO_2_] are presented in red and blue dashed lines, respectively. (Right) Yearly temperature (in $^{\circ}$C) and water deficit (in mm H_2_O), as PET-P (potential evapotranspiration minus rainfall), averaged over the wheat growing period and the shared wheat-growing area of each of the top-twelve world’s wheat producers. decadal trends in temperature (red) and PET-P (blue) are presented.

Supplementary Figure 2. The growing area of wheat relative to other crops (croplands) and total agricultural area, including non-cereals (agriculture), per country in 2019.

Supplementary Figure 3. Changes in relative wheat to croplands area per country from 1961 to 2019 (in %).


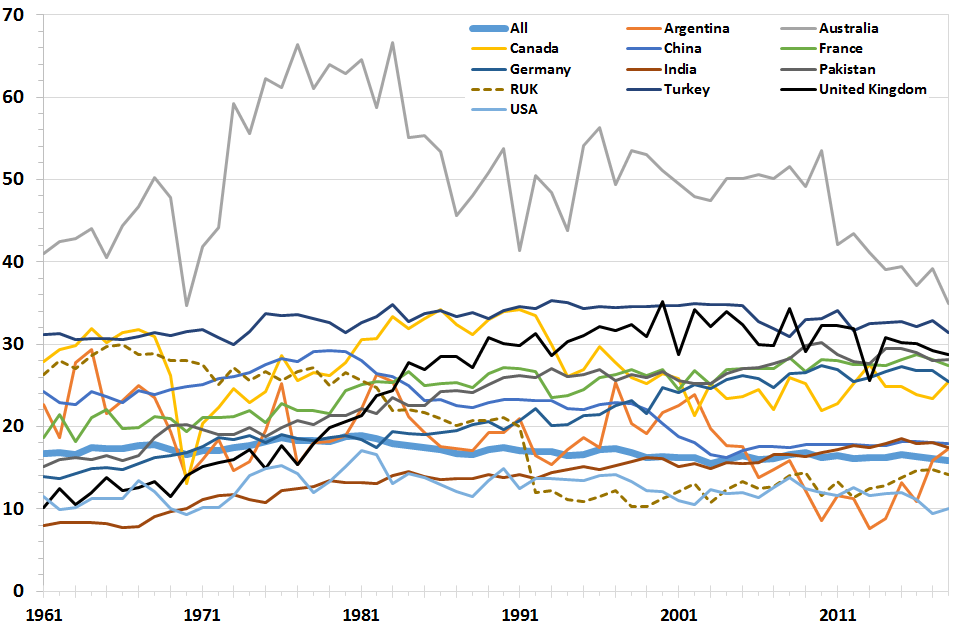


**Supplementary Tables**

Supplementary Table 1. Growing season periods of the top twelve global wheat producers as well as the harvested area and total production for 2019, and whether there is or has been an open-top chamber (OTC) or free-air CO_2_ enrichment (FACE) experiment conducted on wheat in the country/region.

| Country | Wheat growing season (months) | Harvest period (months) | Harvested Area (hectares) | Wheat Production (Mega tons) | FACE/OTC  wheat |
| --- | --- | --- | --- | --- | --- |
| Argentina | Jun-Oct | Nov-Jan | 6,050,953 | 19,459,727 | No |
| Australia | Apr-Oct | Nov-Dec | 10,402,271 | 17,597,561 | **Yes** |
| Canada | May-Aug | Sep | 9,655,600 | 32,347,900 | No |
| China (winter wheat) | Sep-May | May-Jun | 23,732,560 | **133,601,131** | **Yes** |
| France | Oct-Jun | Jul-Aug | 5,244,250 | 40,604,960 | No |
| Germany | Oct-Aug | Aug | 3,118,100 | 23,062,600 | **Yes** |
| India | Oct-Feb | Mar-May | 29,318,790 | 103,596,230 | **Yes** |
| Pakistan | Oct-Mar | Apr-Jun | 8,677,730 | 24,348,983 | No |
| RUK (winter wheat) | Oct-Jul | Jul-Aug | **45,797,858** | 114,119,615 | No |
| Turkey | Sep-Jun | Jun-Aug | 6,831,854 | 19,000,000 | No |
| United Kingdom | Oct-Jul | Aug | 1,816,000 | 16,225,000 | No |
| USA (HRS) | Apr-Jul | Aug-Sep | Total USA: | | **Yes** |
| USA (HRW) | Sep-Apr | May-Aug | 15,039,090 | 52,257,620 |  |

Supplementary Table 2. Summary statistics.

|  | Obs | Mean | Std. dev. | Min | Max |
| --- | --- | --- | --- | --- | --- |
| [CO_2_] | 767 | 356.32 | 27.51 | 315.93 | 413.42 |
| P_CV_ | 767 | 0.577 | 0.278 | 0.045 | 1.866 |
| PET–P | 767 | 155 | 192 | -391 | 548 |
| *T*_mean_ | 767 | 11.082 | 4.698 | -1.353 | 20.700 |
| *T*_max_ | 767 | 15.944 | 6.196 | 3.507 | 28.089 |
| *T*_min_ | 767 | 5.275 | 3.933 | -6.213 | 13.312 |

Supplementary Table 3. Correlation of climate variables and [CO_2_]

|  | [CO_2_] | P_CV_ | PET–P | *T*_avg_ | *T*_max_ | *T*_min_ |
| --- | --- | --- | --- | --- | --- | --- |
| [CO_2_] | 1.0000 |  |  |  |  |  |
| P_CV_ | -0.0336 | 1.0000 |  |  |  |  |
| PET–P | 0.0467 | 0.2910^*^ | 1.0000 |  |  |  |
| *T*_mean_ | 0.0755^*^ | 0.3750^*^ | 0.6268^*^ | 1.0000 |  |  |
| *T*_max_ | 0.0480 | 0.4628^*^ | 0.5170^*^ | 0.8258^*^ | 1.0000 |  |
| *T*_min_ | 0.0951^*^ | 0.3129^*^ | 0.4609^*^ | 0.9565^*^ | 0.7356^*^ | 1.0000 |

Note: Statistical significance is given by ^*^*p*<0.05.

Supplementary Table 4. Same as Table 1 in main text but excluding India from the analysis.

| Independent variable:  Log(Yield) | **T** | **T + W** | **T + W + C** | **T + W + C** + interactions) |
| --- | --- | --- | --- | --- |
| *T*_mean_ | -0.0099^***^ | -0.0055^*^ | -0.010^***^ | -0.011^***^ |
|  | (0.0030) | (0.0035) | (0.004) | (0.004) |
| *T*_max_ | 0.00016 | -0.00008 | 0.00038 | 0.0004 |
|  | (0.00057) | (0.00058) | (0.00058) | (0.0006) |
| *T*_min_ | 0.0024 | 0.00021 | 0.0024 | 0.0024 |
|  | (0.0022) | (0.0023) | (0.0024) | (0.0023) |
| Rain distribution |  | -0.0013 | 0.0004 | 0.0008 |
|  |  | (0.0099) | (0.0098) | (0.0099) |
| Water deficit (PET-P) |  | -0.00004^**^ | -0.00006^***^ | -0.00007^***^ |
|  |  | (0.00002) | (0.00002) | (0.00002) |
| [CO_2_] |  |  | 0.257^***^ | 0.2678^***^ |
|  |  |  | (0.078) | (0.0794) |
| *T*_mean_ $\times$ [CO_2_] |  |  |  | -0.029 |
|  |  |  |  | (0.079) |
| *T*_max_ $\times$ [CO_2_] |  |  |  | 0.0109 |
|  |  |  |  | (0.0162) |
| *T*_min_ $\times$ [CO_2_] |  |  |  | 0.0994 |
|  |  |  |  | (0.0613) |
| Rain distribution $\times$ [CO_2_] |  |  |  | 0.0636 |
|  |  |  |  | (0.2576) |
| Water deficit $\times$ [CO_2_] |  |  |  | 0.0008^*^ |
|  |  |  |  | (0.0004) |
| Adj. Rsq | 0.9656 | 0.9658 | 0.9667 | 0.9678 |
| RMSE | 0.0478 | 0.0476 | 0.0473 | 0.0461 |
| N | 708 | 708 | 708 | 767 |

Notes: T is the model with temperature effects only, T + W is the model that includes the effect of water (rain distribution along the season and water depletion as PET – P), T + W + C is the model including also the CO_2_ effect and T + W + C + interactions model includes the interactions among [CO_2_] and climate variables. Standard errors are displayed in parenthesis. Dependent variable is the logarithm of wheat yield. Rain distribution and CO_2_ in the models are the logarithm of the coefficient of variation in monthly rain amount (standard deviation divided by the mean) and the logarithm of the mean atmospheric CO_2_ concentration (in ppmv) during the growing period. Stars indicate statistical significance: ^*^*p*$\leq$0.1, ^**^*p*$\leq$0.05, ^***^*p*$\leq$0.01.

Supplementary Table 5. Same as Table 1 but using linear Yield instead of Log(Yield).

| Independent variable:  Yield | **T** | **T + W** | **T + W + C** | **T + W + C** + interactions) |
| --- | --- | --- | --- | --- |
| *T*_mean_ | -0.1023^***^ | -0.1100^***^ | -0.066 | -0.1461^***^ |
|  | (0.0345) | (0.0400) | (0.043) | (0.0326) |
| *T*_max_ | -0.1030^**^ | -0.0113^*^ | -0.0166^**^ | -0.0230^***^ |
|  | (0.0065) | (0.0067) | (0.0067) | (0.0051) |
| *T*_min_ | 0.2108^***^ | 0.2121^***^ | 0.1835^***^ | 0.1833^***^ |
|  | (0.0265) | (0.0265) | (0.0272) | (0.0207) |
| Rain distribution |  | 0.022 | 0.1497 | 0.1083 |
|  |  | (0.082) | (0.1069) | (0.0636) |
| Water deficit (PET-P) |  | -0.0008^***^ | -0.0006^***^ | -0.0005^***^ |
|  |  | (0.0001) | (0.0002) | (0.0002) |
| [CO_2_] |  |  | 0.0236^***^ | 0.0268^***^ |
|  |  |  | (0.0058) | (0.0045) |
| *T*_mean_ $\times$ [CO_2_] |  |  |  | -0.0030^***^ |
|  |  |  |  | (0.0007) |
| *T*_max_ $\times$ [CO_2_] |  |  |  | -0.0002 |
|  |  |  |  | (0.0002) |
| *T*_min_ $\times$ [CO_2_] |  |  |  | 0.0041^***^ |
|  |  |  |  | (0.0007) |
| Rain distribution $\times$ [CO_2_] |  |  |  | -0.0087^***^ |
|  |  |  |  | (0.0022) |
| Water deficit $\times$ [CO_2_] |  |  |  | 0.00005^***^ |
|  |  |  |  | (0.000005) |
| Adj. Rsq | 0.9197 | 0.9274 | 0.9219 | 0.9212 |
| RMSE | 0.5539 | 0.4106 | 0.5461 | 0.4611 |
| N | 767 | 767 | 767 | 767 |

Notes: Year was included in all models. T is the model with temperature effects only, T + W is the model that includes the effect of water (rain distribution along the season and water depletion as PET – P), T + W + C is the model including also the CO_2_ effect and T + W + C + interactions model includes the interactions among [CO_2_] and climate variables. Standard errors are displayed in parenthesis. Dependent variable is the logarithm of wheat yield. Rain distribution and CO_2_ in the models are the logarithm of the coefficient of variation in monthly rain amount (standard deviation divided by the mean) and the logarithm of the mean atmospheric CO_2_ concentration (in ppmv) during the growing period. Stars indicate statistical significance: ^*^*p*$\leq$0.1, ^**^*p*$\leq$0.05, ^***^*p*$\leq$0.01.

**References**

1. Abatzoglou, J. T., Dobrowski, S. Z., Parks, S. A. & Hegewisch, K. C. TerraClimate, a high-resolution global dataset of monthly climate and climatic water balance from 1958–2015. *Sci. Data* **5**, 170191 (2018).

2. Ainsworth, E. A. & Long, S. P. 30 years of free‐air carbon dioxide enrichment (FACE): What have we learned about future crop productivity and its potential for adaptation? *Glob. Chang. Biol.* gcb.15375 (2020) doi:10.1111/gcb.15375.

3. Tausz-Posch, S., Norton, R. M., Seneweera, S., Fitzgerald, G. J. & Tausz, M. Will intra-specific differences in transpiration efficiency in wheat be maintained in a high CO_2_ world? A FACE study. *Physiol. Plant.* **148**, 232–245 (2013).

4. Fitzgerald, G. J. *et al.* Elevated atmospheric [CO_2_] can dramatically increase wheat yields in semi-arid environments and buffer against heat waves. *Glob. Chang. Biol.* **22**, 2269–2284 (2016).

5. O’Leary, G. J. *et al.* Response of wheat growth, grain yield and water use to elevated CO_2_ under a Free-Air CO2 Enrichment (FACE) experiment and modelling in a semi-arid environment. *Glob. Chang. Biol.* **21**, 2670–2686 (2015).

6. Zhu, X.-G. & Long, S. P. Can Increase in Rubisco Specificity Increase Carbon Gain by Whole Canopy? A Modeling Analysis BT - Photosynthesis in silico: Understanding Complexity from Molecules to Ecosystems. in (eds. Laisk, A., Nedbal, L. & Govindjee) 401–416 (Springer Netherlands, 2009). doi:10.1007/978-1-4020-9237-4_17.

7. Cai, C. *et al.* Responses of wheat and rice to factorial combinations of ambient and elevated CO_2_ and temperature in FACE experiments. *Glob. Chang. Biol.* **22**, 856–874 (2016).

8. Högy, P., Zörb, C., Langenkämper, G., Betsche, T. & Fangmeier, A. Atmospheric CO_2_ enrichment changes the wheat grain proteome. *J. Cereal Sci.* **50**, 248–254 (2009).

9. Högy, P. *et al.* Grain quality characteristics of spring wheat (Triticum aestivum) as affected by free-air CO_2_ enrichment. *Environ. Exp. Bot.* **88**, 11–18 (2013).

10. Weigel, H.-J. & Manderscheid, R. Crop growth responses to free air CO_2_ enrichment and nitrogen fertilization: Rotating barley, ryegrass, sugar beet and wheat. *Eur. J. Agron.* **43**, 97–107 (2012).

11. Manderscheid, R., Dier, M., Erbs, M., Sickora, J. & Weigel, H. J. Nitrogen supply – A determinant in water use efficiency of winter wheat grown under free air CO_2_ enrichment. *Agric. Water Manag.* **210**, 70–77 (2018).

12. Dier, M. *et al.* Effects of free air carbon dioxide enrichment (FACE) on nitrogen assimilation and growth of winter wheat under nitrate and ammonium fertilization. *Glob. Chang. Biol.* **24**, e40–e54 (2018).

13. Zaveri, E. & B. Lobell, D. The role of irrigation in changing wheat yields and heat sensitivity in India. *Nat. Commun.* **10**, 4144 (2019).

14. Singh, S. D. *et al.* Yield response of important field crops to elevated air temperature and CO_2_ level. *Indian J. Agric. Sci.* **83**, 1009–1012 (2013).

15. Kimball, B. A. *et al.* Productivity and water use of wheat under free-air CO_2_ enrichment. *Glob. Chang. Biol.* **1**, 429–442 (1995).

16. Ainsworth, E. A. & Long, S. P. What have we learned from 15 years of free-air CO_2_ enrichment (FACE)? A meta-analytic review of the responses of photosynthesis, canopy properties and plant production to rising CO2. *New Phytol.* **165**, 351–372 (2005).
